# Anaesthetic management of difficult airway due to retropharyngeal abscess

**DOI:** 10.4103/0019-5049.65376

**Published:** 2010

**Authors:** Manjula Sudhakar Rao, YK Linga Raju, PN Vishwanathan

**Affiliations:** Department of Anaesthesiology, JSS Medical College, Mysore, India

**Keywords:** Difficult airway, difficult intubation, retropharyngeal abscess

## Abstract

A one-and-half-year old girl weighing 7.5 kg presented with a history of neck swelling, difficulty in swallowing and breathing. She was posted for incision and drainage on an emergency basis. Diagnosis was confirmed by neck X-ray and computed tomography scan as retropharyngeal abscess. Here we present the successful anaesthetic management of this child at JSS Medical College Hospital, Mysore.

## INTRODUCTION

Retropharyngeal abscess poses a great challenge to the anaesthesiologist due to difficult airway as a result of the disease process. In this report we present a case of retropharyngeal abscess in a child, which was drained under general anaesthesia successfully without any complications.

## CASE REPORT

A one-and-half-year-old girl weighing 7.5 kg was admitted with a history of fever and cough since 15 days, neck swelling and difficulty in swallowing and mouth opening since 1 week. Diagnosis of retropharyngeal abscess was made using neck X-ray and computed tomography (CT) scan.

**Figure 1:**
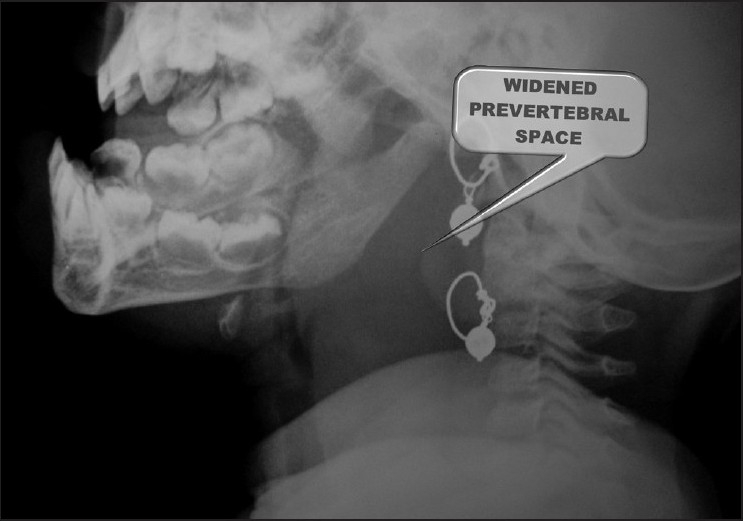
Widened prevertebral soft tissue shadow in X-ray neck lateral view

**Figure 2:**
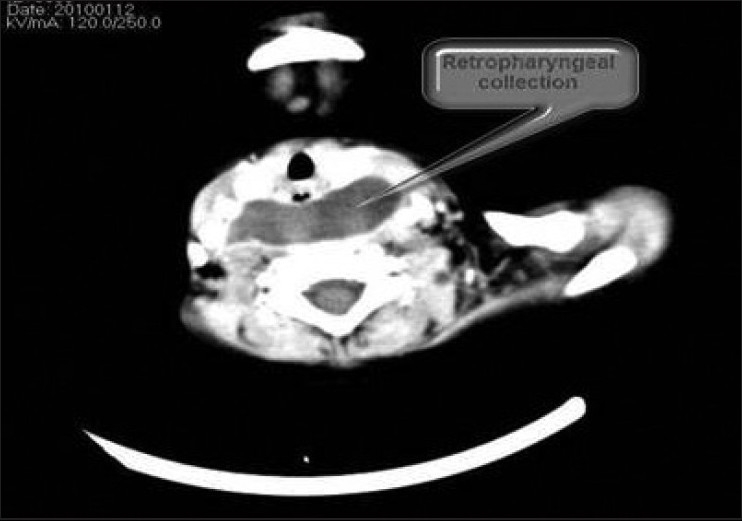
CT neck showing prevertebral collection

The X-ray showed widened prevertebral soft tissue shadow [Figure 1]. CT scan showed fairly large collection with enhancing thin walls in the prevertebral region, extending from base of skull to carina measuring 44 × 20 × 105 mm, suggestive of an abscess [Figure 2]. Laryngeal airways were clear. Laryngeal cartilage and hyoid bone were intact. Carotid arteries and jugular veins were found to be normal. Trachea and main bronchi were clear. No hilar or mediastinal lymphadenopathy was present. Cardia, pericardium and great vessels were normal. Lung fields were clear, and there was no pleural effusion.

Investigations were within normal limits except for raised erythrocyte sedimentation rate (ESR) and leucocytosis. The patient was put on IV cefotaxime 250mg twice daily (BD) and diclofenac suppository. The patient was taken up for intraoral drainage on emergency basis.

Preanaesthetic evaluation was done on the patient She was febrile. There was a neck swelling on the left side. Her pulse was 140 beats/minute, and the SPO_2_ was 98% with room air. Her mouth opening was restricted to one finger. She had difficulty in closing her mouth as well. Mallampati score could not be assessed. Only the upper and lower central incisors were present. Cervical spine was normal.

The respiratory system had the following features. Trachea was central. Air entry was equal, bilateral crepitations and occasional ronchi were present. Rest of the examination was unremarkable.

In view of the anticipated difficult mask ventilation and intubation, all equipments for difficult airway management were kept ready, including that for emergency tracheostomy.

In the operation theatre, IV line was secured with 22-Gauge cannula connected to isolyte P solution. Heart rate, oxygen saturation, respiratory rate and electrocardiogram (ECG) were monitored. Head low position was given to prevent aspiration of abscess contents. The patient was premedicated with inj. midazolam 0.5 mg, inj. pentazocin 4 mg and inj. hydrocortisone 50 mg. In view of the anticipated difficult mask ventilation and intubation, an inhalational induction was planned. Preoxygenation was done for 3 minutes, and anaesthesia was induced with incremental doses of halothane in a mixture of 50% oxygen and 50% nitrous oxide. Under deep inhalational anaesthesia, direct laryngoscopy was done with external laryngeal manipulation to visualise the vocal cords. As Cormach-Lehane grade was 3, i.e. only tip of the epiglottis was visualised, 3.5 mm portex uncuffed endotracheal tube was intubated in the second attempt. The tube was fixed after confirming bilateral air entry. There was pus leak due to trauma during larygoscopy and intubation. After thorough suctioning, throat pack was done to prevent aspiration of blood, pus and secretions. Anaesthesia was maintained with halothane in oxygen and nitrous oxide.

**Figure 3:**
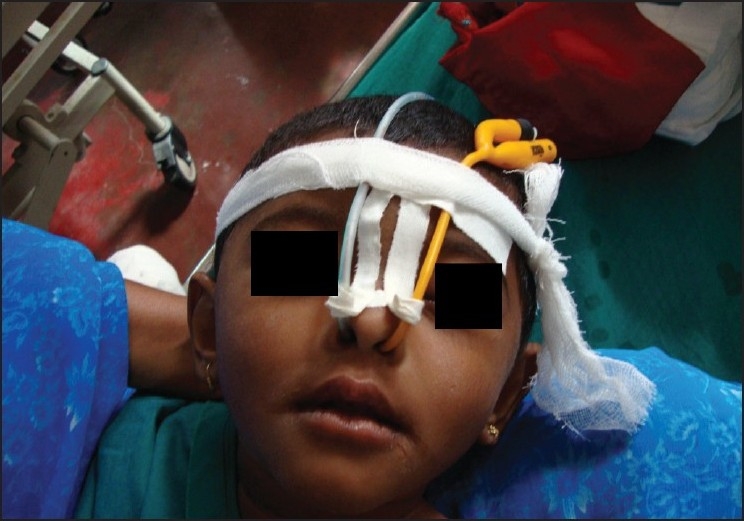
Postoperative picture

**Figure 4:**
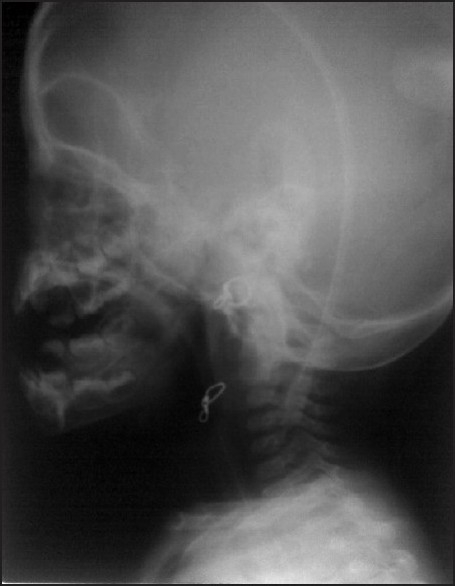
Postoperative neck X-ray lateral view

Inj. atracurium 0.5 mg/kg was given. The patient was ventilated manually throughout the procedure. Intraoral drainage of the abscess was done and 10F size Foleys catheter was placed in the abscess cavity and the bulb inflated with 3ml distilled water [Figures 3 and 4]. Nasogastric tube was inserted. After the patient’s adequate breathing efforts were confirmed, she was reversed using 0.05 mg/kg neostigmine, 0.02 mg/kg atropine, and extubated. Postoperatively, the patient was conscious, alert, breathing adequately, maintaining saturation in room air and haemodynamically stable. The patient was shifted to postoperative ward.

## DISCUSSION

Retropharyngeal abscess is collection of pus in the retropharyngeal space which extends from base of the skull superiorly to the mediastinum inferiorly up to T_1_ level. Anteriorly it is bounded by the posterior pharyngeal wall and posteriorly by the alar fascia. Laterally it is continuous with the parapharyngeal space.[1] It occurs most commonly in childhood. Exactly 50% of patients are younger than 3 years and 70% are younger than 6 years of age.[2] Male: female ratio is 2:1.[2] It occurs due to suppuration of retropharyngeal lymph nodes following an upper respiratory infection in children. It may also be caused secondary to foreign body or scope trauma to the posterior pharyngeal wall. In adults, it is usually secondary to caries of cervical spine.[3]

The clinical features are discussed as follows. It begins insidiously with upper respiratory or ear infection. It is usually associated with constitutional symptoms. Neck stiffness, trismus and mild torticolis may also be present. Infants and children rapidly develop airway compromise and stridor.[1,4] Most of the abscesses are polymicrobial[5] with predominant organisms being *Staphylococcus* aureus and group A *Streptococcus*.[6]

Drainage of the abscess with broad spectrum antibiotics is the treatment of choice.[1,6] Intraoral drainage is preferred if the abscess is confined above the level of hyoid bone. If it extends below the level, it should be drained externally.[1]

Its anaesthetic implications are as follows. The patient is often dehydrated, that results in electrolyte imbalance and metabolic derangements due to poor oral intake.[7] The paitent may be septicemic. If the presentation is delayed, there may be other complications like empyema or mediastinitis.[6] Difficulty in airway management is the major concern. Tracheal intubation is challenging due to distorted airway anatomy, oedema and decreased mouth opening. In early stages, induction of general anaesthesia reduces trismus; however, in later stages induction may precipitate a cannot ventilate, cannot intubate situation.[8] The vocal cords may be difficult to visualise due to swollen pharyngeal wall, airway oedema and laryngeal displacement.[6] Another concern is rupture of abscess and aspiration of the contents during larygoscopy and intubation, which should be gentle to prevent this complication. Thorough throat packing should be done if uncuffed tube is used.[6]
